# Annotations

**Published:** 1911-08-19

**Authors:** 


### ANNOTATIONS.

### Accidental Death.

Accidental death was the verdict given upon a boy aged eleven years who died of abscess of the hip which was said to follow a fall. The question of the part played in disease by a blow may often be extremely difficult to decide. How great the difference of opinion may be with regard to the nature of some cases of nervous disease following injury is well known. In such cases the simulation of organic disease by those described as functional adds to the difficulty. Where, however, some form of inflammation is associated with a history of a blow or a fall, the evidence requires to be very clear as to the time and immediate results of the injury before much stress can be laid upon the deleterious influence of the blow. This is especially the case with children, where falls and blows are frequent. Yet undoubtedly injury may give rise to both acute and chronic disease. In some cases the nature of the association is obscure, but where acute inflammation, such as acute periostitis and osteomyelitis, follows injury the influence of injury is possibly more or less direct. Sufficient damage may be produced in the periosteum or sufficient blood poured out to form a resting place and point of attack for the micro-organisms of the disease. Yet allowing this to be true we must suppose that the micro-organisms were in the blood at the time of the injury or entered very closely after. Since we know micro-organisms to be the cause of the disease it is scarcely possible to arrive at any other conclusion. A tuberculous abscess of the hip might also follow some time after injury, but such an abscess, if terminating fatally, is scarcely likely to lead to an inquest and give rise to the verdict " Accidental death."

### The Growth of the "Moderate Drinker."

The Blue-book just issued, containing the Report for the year 1909 of the Inspector under the Inebriate Acts, is of considerable interest, seeing that the study of alcoholism in its relations to the community is now much to the fore. A conjecture that will attract some attention refers to the proportion of moderate drinkers; this is put at 980 per thousand persons, while free drinkers are given as 17.5 to 18, and the habitual drunkards as the remaining 2 to 2.5 per 1,000. Of those who become habitual drunkards it is surmised that 97 or 98 per cent. die in workhouses, prisons or asylums, or remain drunkards to the last. The prospects of benefit from treatment are therefore very dark, and it is pointed out that for the drunkard who is poor there is practically no opportunity of institutional treatment, while for the destitute there is little chance unless they become criminal. The Report suggests that the best means of dealing with the offences of occasional drunkards is to mete out sharp punishment in order to emphasise the necessity for self-control. In regard to the association of recognised forms of mental defect or disease with habitual drunkenness, it is concluded that this association is not definite enough to justify the assertion that all inebriates are more or less insane. But it is probable that even the most mentally sound inebriates are the victims of a constitutional peculiarity which cannot yet be defined or located, although the evidences of its existence are none the less definite on that account. It is almost certain that this peculiarity is an inherited diathesis in most cases.

### A New Patent-medicine Stamp.

Attention has often been called to the moral effect, on the minds of purchasers of patent medicines, of the Government stamp which is affixed to the packages. It has been thought that among certain classes this stamp has not seldom been taken to mean an implied Government guarantee, notwithstanding the presence of words on the stamp directly contrary. As a result of some ventilation of the subject in Parliament, a new stamp has now been issued by Somerset House in which the design rather more effectively emphasises the absence of any such guarantee, while instructions are also given on the stamp as to the manner in which it is to be affixed.

### Bedrooms in Basements in Marylebone.

In the lay press " A Doctor's Daughter " drew attention last week to a grievance arising in connection with the regulations governing the use of basements as sleeping places. She refers to the consequences likely to follow if the regulations authorised by the Local Government Board are pressed in such a borough as Marylebone. It will be recalled that under the Public Health Acts and under the Housing and Town Planning Act of 1909 a room habitually used as a sleeping place, the surface of the floor of which is more than 3 ft. below the surface of the road, shall be deemed unfit for human habitation, unless the height of the room be at least 7 ft. This beneficent clause seems, according to the writer of the letter, to have been applied to those houses in which the menservants have been in the habit of sleeping downstairs near their silver cupboards and away from the young maids of the establishment. It is difficult to suppose that such an application was intended by the framers of the measure, seeing that such basements are generally well drained and ventilated. As the letter points out, no little hardship is likely to follow if servants are not to be allowed to sleep in these basements, for the more limited accommodation will mean the employment of fewer domestics. It is probably only intended to apply the clause to a limited number of cases, for we imagine there cannot be many basements, in houses of that class in the West End, which are under 7 ft. in height, a point which the writer of the letter does not refer to. The effect of the clause on " cellar dwellings " in general has been entirely in the direction of improvement from the public health point of view, and if even the master and mistress are not allowed to sleep in their own basements, as they might feel inclined to do in order to make room upstairs for their servants, it is surely all for their own good.

